# Restoration of Type 17 immune signaling is not sufficient for protection during influenza-associated pulmonary aspergillosis

**DOI:** 10.3389/fimmu.2025.1529849

**Published:** 2025-01-30

**Authors:** Aijaz Ahmad, Ravineel B. Singh, Kara L. Nickolich, Matthew J. Pilewski, Caden Ngeow, Kwame Frempong-Manso, Keven M. Robinson

**Affiliations:** Division of Pulmonary, Allergy, Critical Care and Sleep Medicine, Department of Medicine, University of Pittsburgh, Pittsburgh, PA, United States

**Keywords:** interleukin-17, IAPA, antimicrobial peptides, *Aspergillus fumigatus*, interleukin-22 (IL-22)

## Abstract

**Introduction:**

Influenza-associated pulmonary aspergillosis (IAPA) is a severe complication of influenza infection that occurs in critically ill patients and results in higher mortality compared to influenza infection alone. Interleukin-17 (IL-17) and the Type 17 immune signaling pathway cytokine family are recognized for their pivotal role in fostering protective immunity against various pathogens. In this study, we investigate the role of IL-17 and Type 17 immune signaling components during IAPA.

**Methods:**

Wild-type mice were challenged with influenza A H1N1 (flu) and then exposed to *Aspergillus fumigatus* ATCC42202 resting conidia on day 6 post-influenza infection, followed by the quantification of cytokines and chemokines at 48 h post-fungal infection.

**Results and discussion:**

The gene and protein expression levels revealed that IL-17 and Type 17 immune cytokines and antimicrobial peptides are downregulated during IAPA compared to mice singularly infected solely with *A. fumigatus*. Restoration of Type 17 immunity was not sufficient to provide protection against the increased fungal burden observed during IAPA. These findings contrast those observed during post-influenza bacterial super-infection, in which restoration of Type 17 immune signaling protects against exacerbation seen during super-infection. Our study highlights the need for future studies to understand the immune mechanisms that increase susceptibility to fungal infection.

## 1 Introduction

Influenza-associated pulmonary aspergillosis (IAPA) is a severe complication of influenza infection that occurs in critically ill patients and results in higher mortality compared to influenza infection alone (1). The pathology of IAPA manifests through the invasive growth of *Aspergillus fumigatus* within the lungs during influenza infection (2). Influenza virus damages the respiratory epithelium, compromising the barrier function and allowing opportunistic fungi such as *A. fumigatus* to invade and colonize the lung tissue. The dysregulated immune state of the host, caused by the viral infection, provides an optimal environment for fungal growth and dissemination.

The interleukin-17 (IL-17) cytokine family is a pivotal component in mediating protective immunity against various pathogens (3). IL-17, primarily produced by T cells including T helper cells (Th17 cells) and γδ T cells, has been implicated in the immune response against extracellular pathogens. IL-17 exerts its immunomodulatory effects by promoting the synthesis of pro-inflammatory molecules, including cytokines, chemokines, and antimicrobial peptides. These molecules collectively facilitate the recruitment and activation of neutrophils and other immune effectors to the sites of infection, thereby augmenting the host defense against invading pathogens (4).

Despite the well-established role of IL-17 in immunity against various pathogens, its specific involvement in the context of IAPA remains understudied. Therefore, understanding the immune response in this context is vital to improve the therapeutic strategies and patient outcomes. Our current study investigates the role of IL-17 and IL-17-related cytokines in IAPA, employing a murine model to delineate their impact on disease pathogenesis and immune responses. Our observations shed light on the complex host immune system that occurs during IAPA, aiming to uncover novel therapeutic avenues and enhance patient outcomes in managing IAPA.

## 2 Materials and methods

### 2.1 Animals

Foremostly, 6- to 8-week-old male C57BL/6 mice were purchased from Taconic Farms (Germantown, NY, USA). The mice were kept in a pathogen-free environment and co-housed in the same facility before the commencement of the studies. All animal studies were performed according to the protocol for the care and use of animals sanctioned by the University of Pittsburgh Institutional Animal Care and Use Committee. All of the studies used age- and sex-matched mice.

### 2.2 Pathogens and super-infection model

Influenza A/PR/8/34 H1N1 was propagated in chicken eggs as previously described (5) or by using Madin–Darby canine kidney (MDCK) cells. The cells were maintained in DMEM with 10% FBS (Bio-Techne, Minneapolis, MN, USA), penicillin (100 U/mL), and streptomycin (100 ug/mL) (Invitrogen, Waltham, MA, USA). The cells were washed with PBS and infected 0.001 MOI of influenza virus A/Puerto Rico/8/1934 (H1N1) in DMEM with 0.2% bovine serum albumin (Invitrogen, Waltham, MA, USA) and 2 μg/mL of L-tosylamido-2-phenyl ethyl chloromethyl ketone (TPCK) (Sigma-Aldrich, MO, USA). The virus containing supernatant was harvested after 72 h, and the viral titer was determined by a standard plaque assay. The mice were infected with 100 PFU of influenza A/PR/8/34 H1N1 (in 50 μL sterile PBS) from a frozen stock or control PBS by oropharyngeal aspiration under light anesthesia with isoflurane. The infected mice were incubated for 6 days, at which time the mice received 2.5 × 10^7^ conidia of *A. fumigatus* ATCC42202 inoculum or PBS control. At 24–120 h post-fungal infection, all of the mice were euthanized for their lungs to be harvested for further studies.

#### 2.2.1 Adenoviral IL-17 infection

The mice were infected with adenovirus expressing IL-17 (2.5 × 10^8^ PFU in 50 µL sterile PBS) or enhanced GFP (EGFP) (1 × 10^10^ PFU in 50 µL sterile PBS) (control) by oropharyngeal aspiration 3 days after the influenza A infection. For adenoviral IL-17, an E1- and E3-deleted adenoviral vector encoding enhanced green fluorescent protein (Ad-eGFP) was constructed *via* Cre-lox recombination as described previously (6, 7) using the reagents provided by S. Hardy (Somatix, Alameda, CA, USA). Briefly, a SnaBI–HpaI fragment was created by inserting the cytomegalovirus promoter, eGFP or mIL-17 cDNA, and SV40 poly(A) sequence into the pAdlox shuttle plasmid. E1-substituted recombinant adenovirus was generated by co-transfection of SfiI-digested pAdlox-EGFP and ψ5 helper virus DNA into the adenoviral packaging cell line CRE8, propagated, and purified as described (7). The E1- and E3-deleted adenovirus encoding mouse interleukin 17 (Ad-mIL-17) was constructed by cloning the mIL-17 cDNA as a SalI–NotI fragment in the pAdlox shuttle plasmid. E1-substituted recombinant adenovirus was then generated by co-transfection as described above.

#### 2.2.2 Administration of recombinant cytokines and antimicrobial peptides

Male C57BL/6 mice, aged 6 to 8 weeks, were infected by oropharyngeal aspiration with 100 plaque-forming units (PFU) of influenza A virus (strain A/PR/8/34 H1N1) in 50 µL of sterile PBS as described above.

The mice were administered with recombinant cytokines IL-17A (2 µg/50 µL), IL-1β (25 ng/50 µL), IL-23 (1 µg/50 µL), and IL/22 (1 µg/50 µL) (R&D Systems, Minneapolis, MN, USA) *via* oropharyngeal aspiration at both 24 h and 2 h prior to *A. fumigatus* infection. The mice were administered with recombinant antimicrobial peptides Reg3β (25 µg/50 µL) and Reg3γ (15 µg/50 µL) (R&D Systems, Minneapolis, MN, USA) *via* oropharyngeal aspiration at 4 h prior to *A. fumigatus* infection. The control mice received an equal volume of PBS without therapeutic agents.

### 2.3 Lung inflammation analysis

After harvesting, the mouse lungs were lavaged with 1 mL of sterile PBS to perform inflammatory cell differential counts using standard cytospin technique. The upper lobe of the right lung was homogenized in sterile PBS for counting fungal colonies and cytokine analysis, conducted either with Lincoplex (Millipore, MO, USA) or ELISA assays (R&D Systems, MN, USA), following the manufacturer’s guidelines. The middle and lower lobes of the right lung were snap-frozen and then homogenized under liquid nitrogen for RNA extraction using the Absolutely RNA Miniprep Kit (Agilent Technologies, TX, USA) and RNeasy^®^ Mini Kit (QIAGEN Gmbh, Hilden, Germany). The concentration and purity of the extracted total RNA were measured with a Nanodrop spectrophotometer. Total RNA extraction was followed by the synthesis of cDNA and was performed using Bio-Rad iScript cDNA synthesis kit (Bio-Rad, USA), following the manufacturer’s instructions. The RNA analysis was carried out *via* standard predesigned Taqman gene expression assays. In each plate, hypoxanthine-guanine phosphoribosyltransferase (*HPRT*) was used as housekeeping gene. Gene expression analysis was performed from two replicate samples. It was calculated using the formula ΔCq = 2^Cq target gene-Cq reference gene^, where the quantitation cycle (Cq) was the average Cq value of the target gene minus the *HPRT* reference gene’s mean.

### 2.4 Flow cytometry

Flow cytometry analysis was conducted on the whole left lung. After harvesting, the left lung underwent collagenase digestion, following a previously described protocol (8). The resulting single-cell preparations were *in vitro* stimulated with PMA (50 ng/mL) and ionomycin (750 ng/mL) for 4 h at 37°C. Subsequently, the cells were stained with antibodies, fixed and permeabilized, and stained with fluorescent-conjugated antibodies (BD Biosciences). The following anti-mouse fluorescently labeled antibodies were used: CD45, γδTCR, and CD4 BD (Bioscience). For detection of the intracellular expression of IL-17A and IL-22, cell suspensions were fixed using Fix and Perm (Invitrogen, Thermo Fisher, CA, USA) and stained with anti-mouse IL-17A and IL-22 (BD Bioscience) for 30 min at room temperature in the dark.

## 3 Results

### 3.1 Type 17 cytokines are inhibited during IAPA

**Figure 1:**
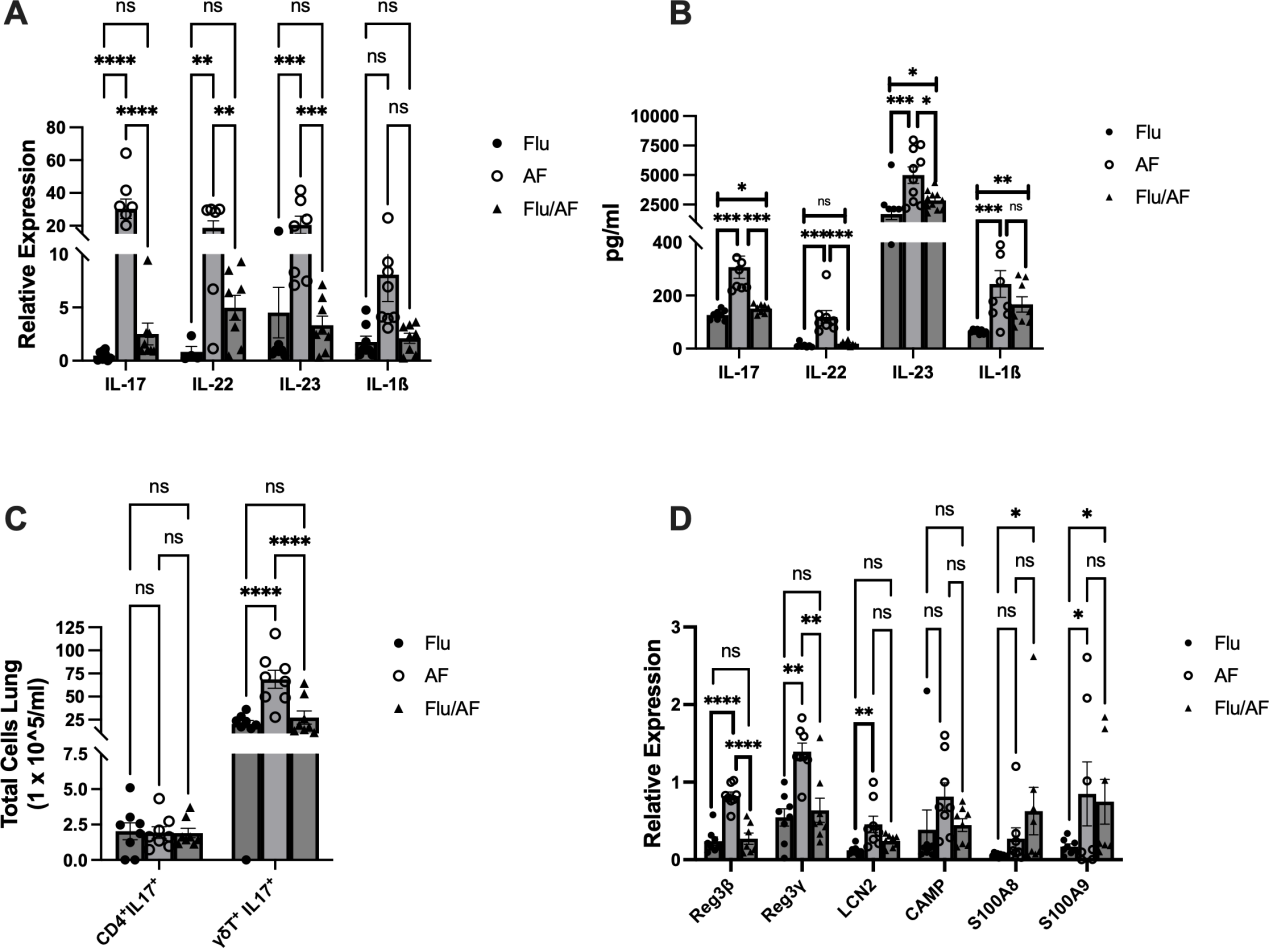
Type 17 immune pathway is downregulated during influenza-associated pulmonary aspergillosis (IAPA). WT mice were infected with influenza A H1N1 PR/8/34 on day 0 and subsequently challenged with 2.5 × 10^7^ *Aspergillus fumigatus* (AF) at 6 dpi. Lung samples were collected at 48 h post-AF challenge. **(A)** IL-17 and related pro-inflammatory cytokine gene expression quantified by RT-qPCR. **(B)** Protein levels of IL-17-related cytokines quantified using Lincoplex assay or ELISA. **(C)** Flow cytometry analysis of IL-17-producing cells, CD4^+^ IL-17^+^ T cells, and γδ T cells. **(D)** IL-17/IL-22-associated antimicrobial peptide gene expression quantified by RT-qPCR. Data were compiled from two independent experiments and are presented as means ± SEM, with statistical significance marked as *p < 0.05, **p < 0.005, ***p < 0.0005, and ****p < 0.0001 by ordinary one-way and two-way ANOVA with Tukey’s multiple-comparisons test.

We have previously published a murine model of IAPA that demonstrates increased morbidity in mice co-infected with influenza and *A. fumigatus*. In this study, we observed the greatest differences in fungal burden between singular *Aspergillus* infection and IAPA at 48 and 72 h post-fungal challenge (9). Type 17 immunity plays a critical role in host defense against *A. fumigatus* and other fungal pathogens (10–14). IL-17 and other Type 17 immune cytokines also play a critical role in the development of bacterial super-infection during influenza (15–17). Therefore, we hypothesized that the Type 17 immune response would play an essential role during IAPA. First, C57BL/6 male mice were challenged with a sublethal dose of influenza A PR/8/34 H1N1 (100 PFU) for 6 days, followed by 2.5 × 10^7^ *A. fumigatus* (ATCC strain 42202) conidia, and IL-17 expression was measured at 24–120 h post-fungal challenge. We observed significant attenuation of IL-17 at 24 and 48 h post-fungal challenge in IAPA mice compared to singular infection with *Aspergillus*. Beyond 48 h post-infection, no significant changes in IL-17 expression were observed, suggesting that the peak differences occur within the first 48 h post-fungal challenge, indicating a critical window of immune response during this period (**Supplementary Figure S1**). Given these findings, the remainder of our experiments were harvested at 48 h post-fungal challenge. To investigate Type 17 immune signaling during IAPA, C57BL/6 male mice were challenged with a sublethal dose of influenza A PR/8/34 H1N1 (100 PFU) for 6 days, followed by 2.5 × 10^7^ *A. fumigatus* (ATCC strain 42202) conidia, and after 48 h, fungal burden was assessed. Mice super-infected with influenza and *A. fumigatus* had a decreased expression of IL-17, IL-22, and IL-23 compared to those infected with *A. fumigatus* alone (**Figure 1A**). In addition to the gene expression changes, protein expression analysis also showed a decreased production of IL-17, IL-22, and IL-23 in super-infected mice (**Figure 1B**). Although the mRNA and protein levels of IL-1β were not significantly decreased during IAPA compared to singular infection with *A. fumigatus*, there was a clear trend toward the inhibition of IL-1β during IAPA. We hypothesized that the downregulation of Type 17 immunity could potentially contribute to increased morbidity, mortality, and fungal burden compared to singular infection with *A. fumigatus* alone.

### 3.2 Decreased IL-17-producing γδ T cells during IAPA

Flow cytometry analysis was conducted to quantify IL-17-producing T cells in our model. We measured IL-17^+^ CD4^+^ T and γδ T cells in our model due to their well-established role as primary producers of IL-17. We observed a notable decrease in the total number of IL-17-producing γδ T cells in super-infected mice compared to singular *A. fumigatus* infection (**Figure 1C**). Comparatively, we observed no difference in the abundance of IL-17^+^ CD4^+^ T cells between the different groups (**Figure 1C**).

### 3.3 Reduced expression of IL-17/IL-22-associated antimicrobial peptides in IAPA

As IL-17 and IL-22 are distinct lineages of Type 17 cells (18), we studied their effector function by examining the gene expression levels of IL-17- and IL-22-associated antimicrobial peptides in our model. The expression levels of *Reg3β* and *Reg3γ* were significantly reduced in super-infected mice compared to those infected solely with *A. fumigatus* (**Figure 1D**).

### 3.4 Restoration of Type 17 immunity does not enhance fungal clearance during IAPA

**Figure 2:**
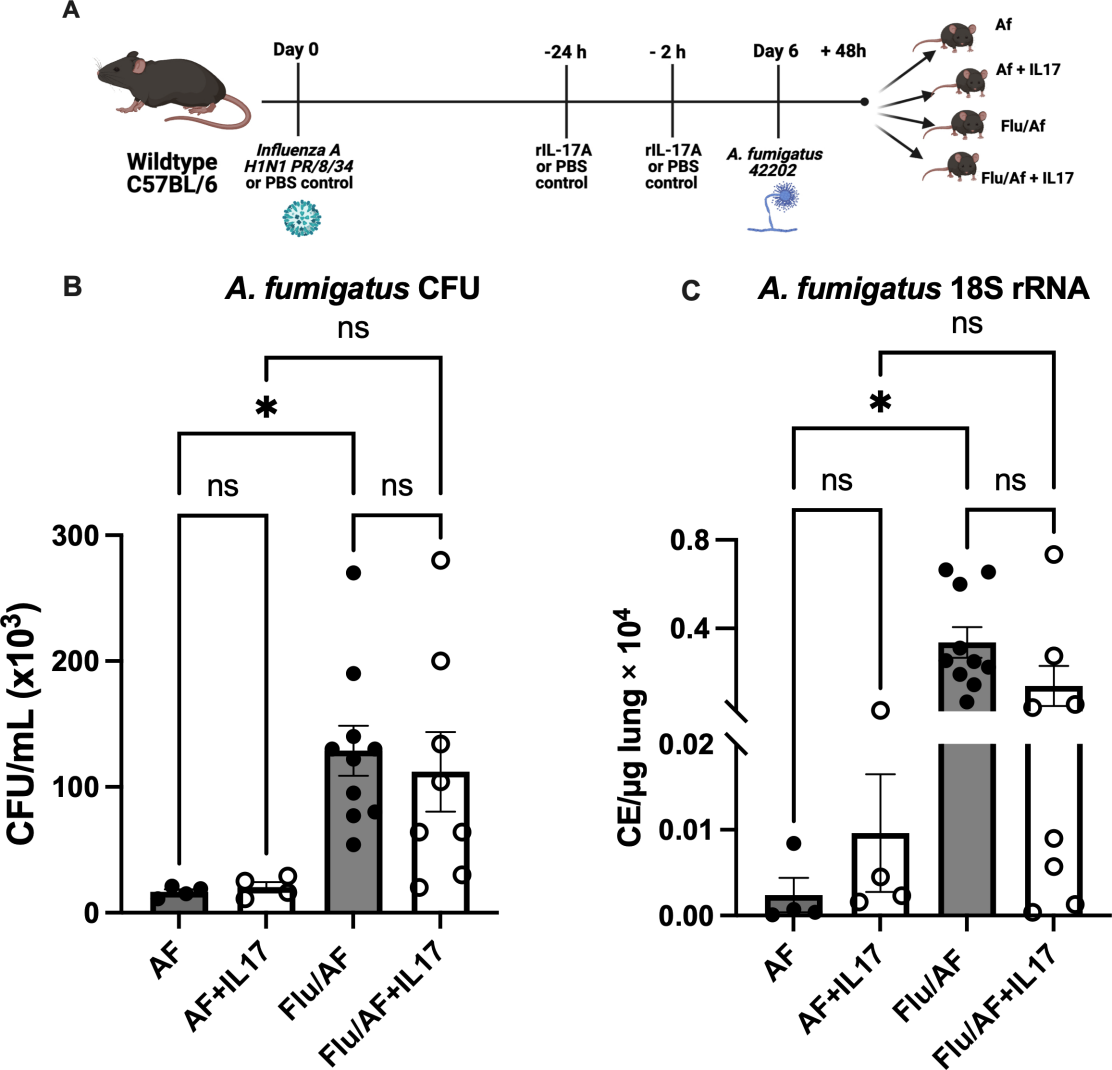
Administration of recombinant IL-17 does not enhance fungal clearance during pulmonary aspergillosis or post-influenza pulmonary aspergillosis. Wild-type (WT) mice were infected intratracheally with 100 PFU of influenza A virus (strain PR/8/34) or PBS (AF) group and maintained for 6 days to establish a viral infection. The mice were then challenged intratracheally with 2.5 × 10^7^ resting conidia of *Aspergillus fumiga*tus (AF) for 48 h to induce invasive pulmonary aspergillosis. At 24 and 2 h prior to fungal infection, the mice were administered externally with recombinant IL-17 or PBS control **(A)**. Lung fungal burden was quantified by enumeration of colony-forming units (CFU) **(B)** and by RT-qPCR targeting *A fumigatus 18S* rRNA levels **(C)**. Data represent mean ± SEM from two independent experiments. Statistical significance was determined using one-way ANOVA followed by Tukey’s *post hoc* test, with significance defined as *p < 0.05.

**Figure 3:**
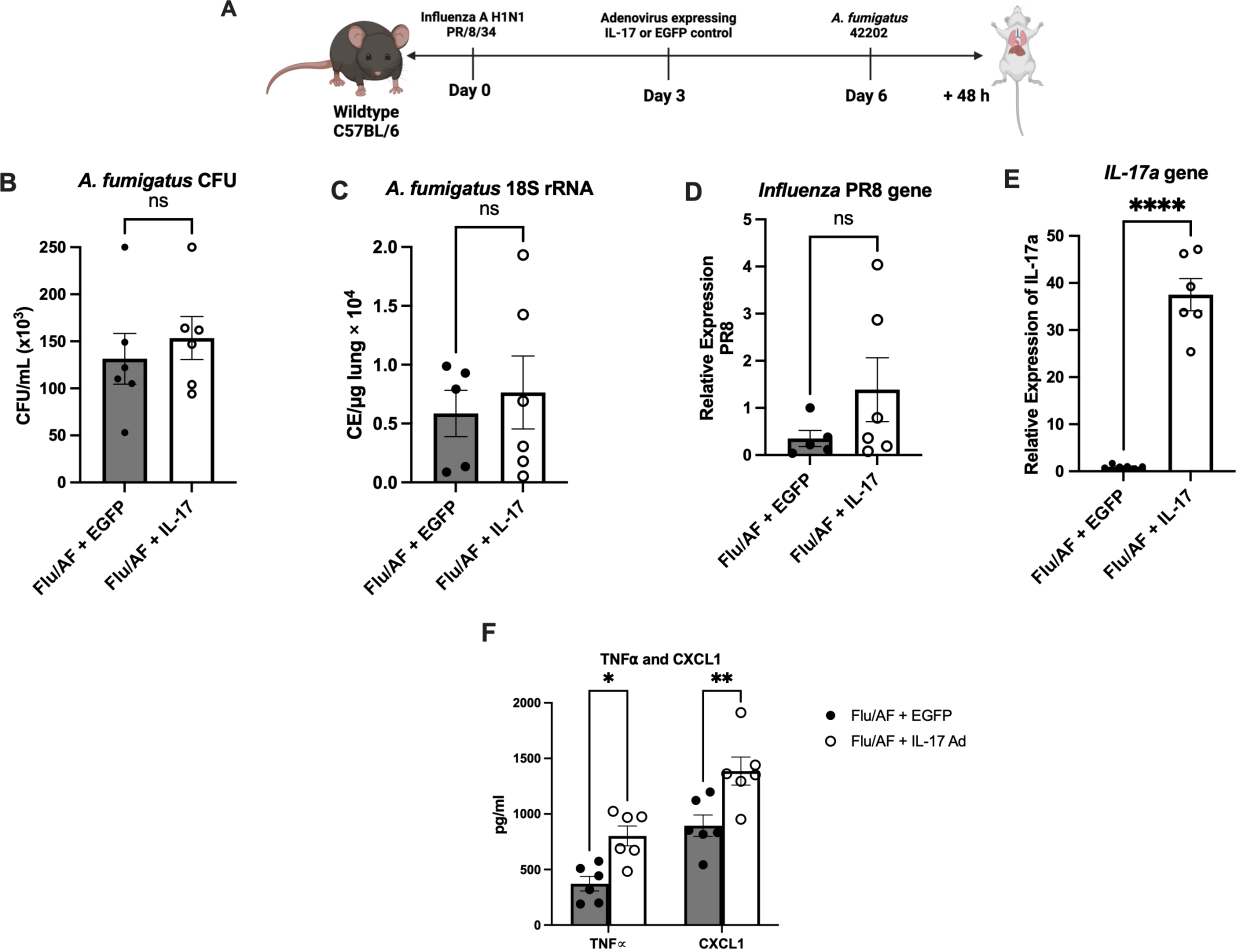
Exogenous administration of IL-17 expressing adenovirus does not provide protection against IAPA. IAPA mice were administered IL-17 expressing adenovirus (IL-17) or control adenovirus on day 3 post-influenza infection **(A)**. Lung fungal burden was measured by CFU **(B)** and by RT-q PCR of *A fumigatus 18S* rRNA levels **(C)**. Influenza matrix protein expression was measured in lung RNA **(D)**. Expression of IL-17a mRNA by RT-qPCR **(E)**. TNFα and CXCL1 cytokines measured by ELISA **(F)**. Data were expressed as mean ± SEM from two independent experiments and were analyzed for statistical significance using ordinary one-way ANOVA followed by Dunnett’s multiple-comparisons test. Significance levels are denoted by *p < 0.05, **p < 0.005 and ****p < 0.0001.

With the observation that Type 17 cytokines and associated antimicrobial peptides were significantly downregulated during IAPA compared to *A. fumigatus* alone, we hypothesized that their restoration could provide protection during IAPA. To test this hypothesis, we administered murine recombinant IL-17 protein during *A. fumigatus* infection and IAPA (**Figure 2A**). Although fungal burden, as measured by CFU and the *A. fumigatus 18S* rRNA gene, was significantly higher in IAPA compared to *A. fumigatus* infection alone, IL-17 administration did not result in significant changes in CFU counts (**Figure 2B**) or *18S* rRNA gene expression (**Figure 2C**) among the treated groups. Given the anticipated role of IL-17 in rescuing Type 17 immunity and potentially overcoming the limitations of recombinant protein administration, such as poor delivery system, rapid clearance, and short half-life, we turned to an adenoviral vector expressing IL-17 in the influenza and *A. fumigatus*-superinfected mice, proposing that it would rescue Type-17 immunity and enhance fungal clearance (**Figure 3A**). Previous studies have demonstrated that an intratracheal administration of IL-17-expressing adenovirus induces a localized release of IL-17 in the murine lung, with levels rising by 24 h, peaking at 72 h, remaining near-peak at 96 h, and persisting at lower levels for up to 7 days (19). In our study, IL-17-expressing or control adenovirus was administered to IAPA mice on day 3 post-influenza infection. Consequently, the IL-17 levels in the lung are expected to be elevated prior to the secondary *Aspergillus* challenge and remain high throughout the 48-h harvest time point. With the overexpression of IL-17, the fungal burden, measured by CFU/mL (**Figure 3B**) and *18S* rRNA gene expression (**Figure 3C**), as well as the influenza viral load (**Figure 3D**), remained unchanged between the two mouse groups. However, the overexpression of IL-17 highly upregulated the levels of IL-17a mRNA (**Figure 3E**) and downstream inflammatory mediators TNFα and CXCL1 during IAPA (**Figure 3F**) (20, 21). Interestingly, the total number of inflammatory cells in the bronchoalveolar lavage fluid and the total number of neutrophils and macrophages measured by cytospin differential also remained unchanged with the upregulation of Type 17 immunity (**Supplementary Figure S2**).

**Figure 4:**
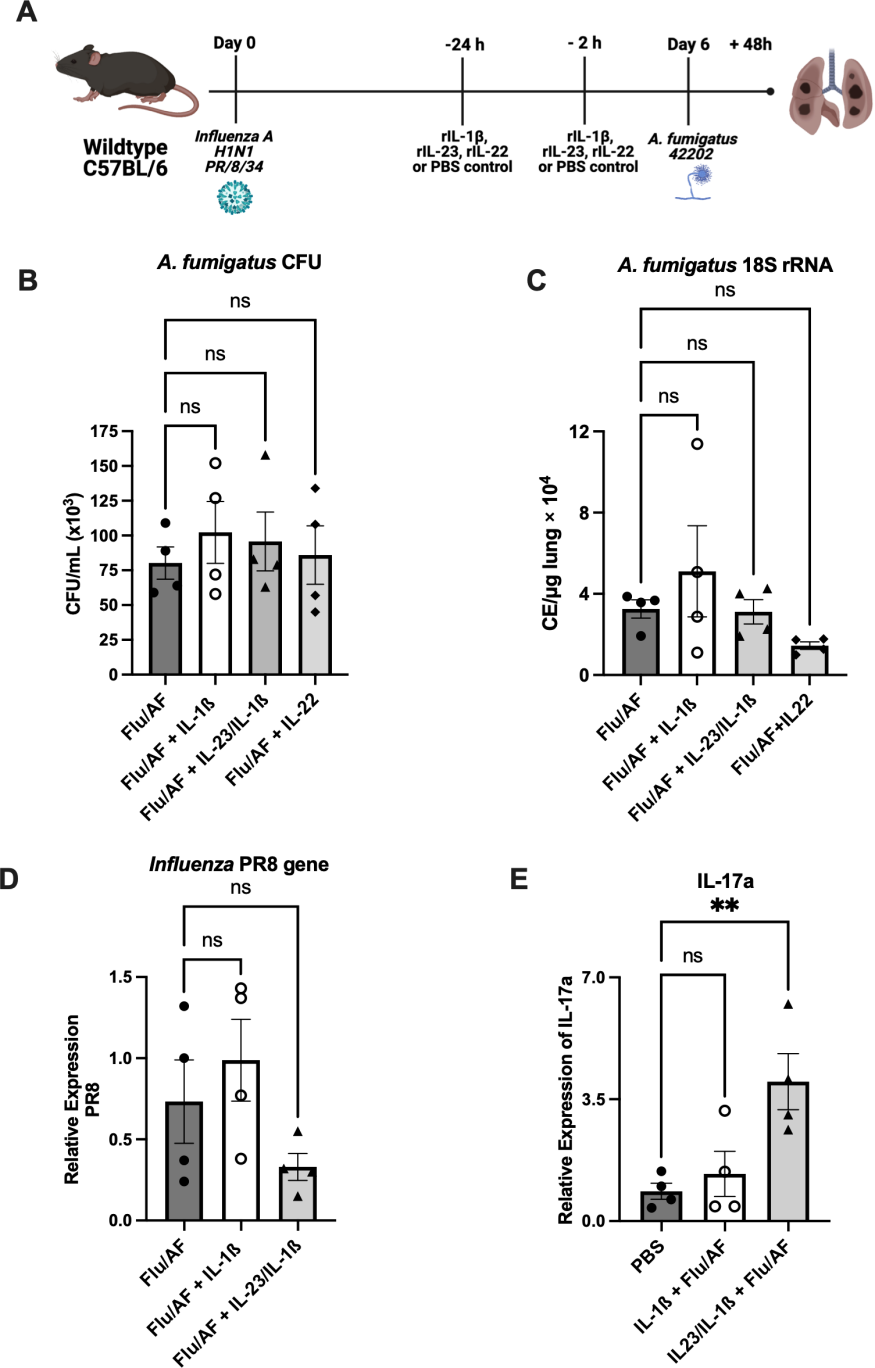
Restoration of Type 17 immune signaling is not sufficient to provide protection during IAPA. IAPA mice were exogenously administered with IL-1β, IL-1β+IL-23, or IL-22 murine recombinant proteins **(A)**. Lung fungal burden was measured by CFU **(B)** and by RT-q PCR of *A fumigatus 18S* rRNA levels **(C)**. Influenza matrix protein expression was measured in lung RNA **(D)**. Expression of IL-17a mRNA by RT-qPCR **(E)**. Data were expressed as mean ± SEM and were analyzed for statistical significance using ordinary one-way ANOVA followed by Dunnett’s multiple–comparisons test. Significance levels are denoted by **p < 0.05.

Next, we restored the components of the Type 17 immune signaling pathway that are both upstream and downstream of IL-17 to determine the effects on fungal clearance. Since IL-1β and IL-23 are known to induce IL-17 production from Th17 and γδ T cells (22, 23), and we observed decreased IL-17 producing cells during IAPA (**Figures 1A, B**), we administered IL-1β or IL-23+IL-1β together for synergistic effects during IAPA (**Figure 4A**). The administration of exogenous IL-1β alone was not able to significantly upregulate IL-17A mRNA expression (**Figure 4E**) in our model; however, treatment with IL-23 and IL-1β together significantly upregulated IL-17 mRNA expression (**Figure 4E**). Despite the increased IL-17A levels observed, the administration of neither IL-1β nor IL-23+IL-1β enhanced fungal clearance, as indicated by unchanged CFU/mL levels (**Figure 4B**) and *18S* rRNA expression (**Figure 4C**). Additionally, viral burden was not altered (**Figure 4D**). We observed no differences in the total cell count of immune cells in bronchoalveolar lavage fluid in mice that received IL-1β or IL-23+IL-1β; however, there were increased numbers of neutrophils in the bronchoalveolar lavage fluid of mice that received IL-1β alone (**Supplementary Figure S3**). IL-22 is another critical cytokine in the Type 17 immune signaling pathway that plays an important role in host defense against various pathogens (24). The administration of exogenous IL-22 did not enhance fungal clearance as no significant changes were observed in the CFU/mL levels (**Figure 4B**) and *18S* rRNA expression (**Figure 4C**) when the mice were administered with recombinant IL-22.

**Figure 5:**
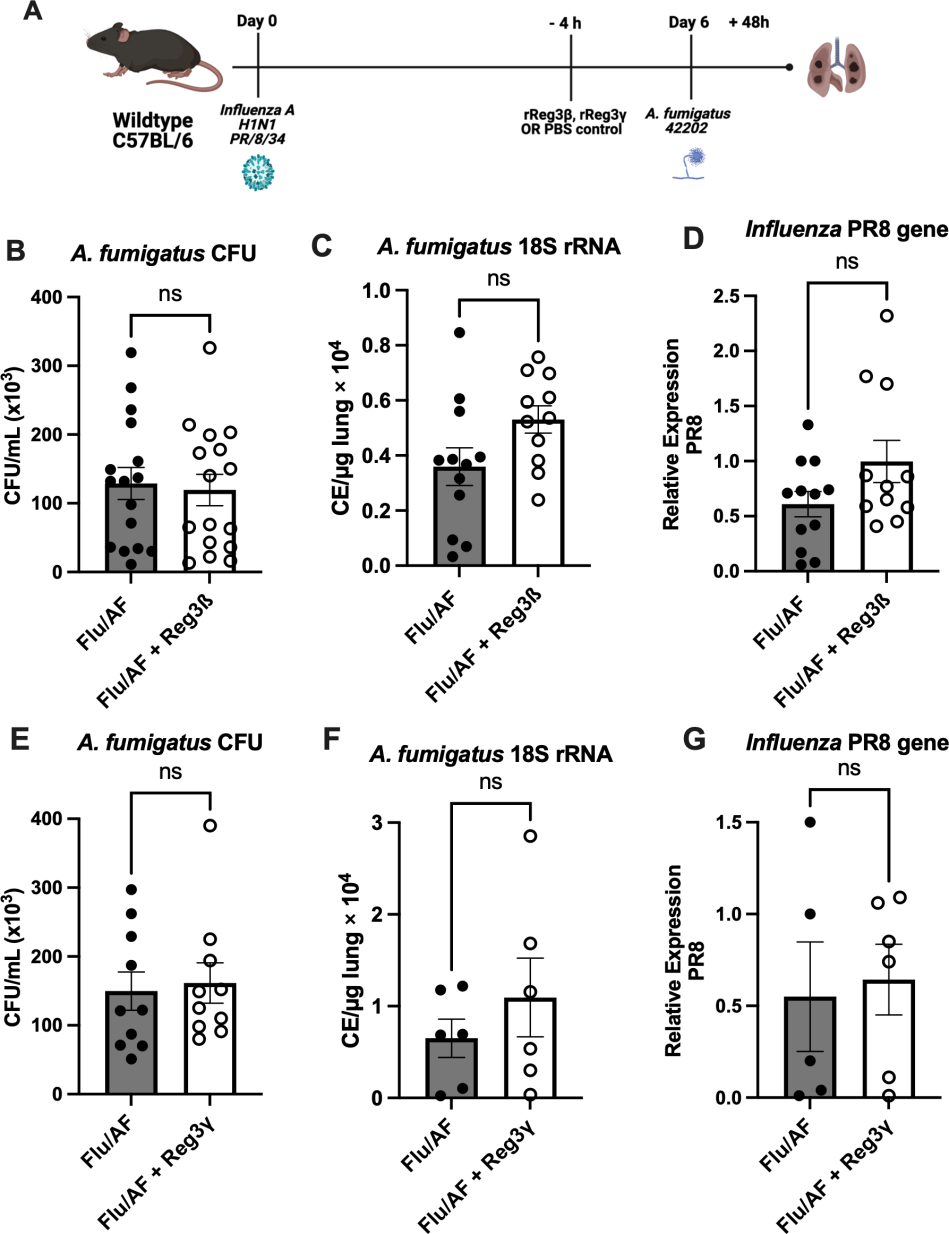
Inefficacy of antimicrobial peptide administration in rescuing phenotypic manifestations. IAPA mice were exogenously administered with antimicrobial peptide Reg3β or Reg3γ **(A)**. Lung fungal burden was measured by CFU **(B)** and by RT-q PCR of *A fumigatus 18S* rRNA levels **(C)**. Influenza matrix protein expression was measured in lung RNA **(D)**. Lung fungal burden was also measured for mice administered with external Reg3γ by CFU **(E)** and by RT-q PCR of *A fumigatus 18S* rRNA levels **(F)**. Influenza matrix protein expression was measured in lung RNA **(G)**. Data were compiled from two independent experiments and expressed as mean ± SEM and were analyzed for statistical significance using *T*-test.

Cytokines IL-17 and IL-22 are known to promote the production of antimicrobial peptides, including Reg3β and Reg3γ, by epithelial cells which act downstream to enhance innate immune responses (24). These peptides are integral in maintaining the mucosal barrier function and providing frontline protection against fungal pathogens in the respiratory tract. Furthermore, Reg3γ is also reported to specifically bind to bacterial and fungal pathogens, including *A. fumigatus* (25), which facilitate direct antimicrobial activity and limit fungal colonization and invasion in the respiratory tract. In line with this, super-infected mice were administered either with Reg3β or Reg3γ to explore possible potential therapeutic interventions (**Figure 5A**). Although both of these peptide levels were reduced in super-infected mice, exogenous administration did not alter fungal or viral clearance (**Figures 5B–G**). Additionally, we observed no difference in the total cell count of immune cells in bronchoalveolar lavage fluid (**Supplementary Figure S3**). Collectively, these results indicate that restoration of IL-17 signaling is not sufficient to restore fungal clearance during IAPA. These findings suggest the involvement of mechanisms beyond Type 17 inhibition, causing decreased fungal clearance during IAPA and highlighting the complexity of IAPA pathogenesis.

## 4 Discussion

Viral–fungal co-infections are increasingly recognized as a significant contributor to high mortality rates during influenza infection, yet our understanding of their pathophysiology and the associated immunological response in the lungs remains limited. Influenza and *A. fumigatus* have individually been studied due to their clinical significance; however, the synergy and complexities that arise when these pathogens co-exist within the same host remain understudied. Invasive pulmonary aspergillosis was classically considered a disease of immunocompromised patients; however, recent clinical observations have reported invasive pulmonary aspergillosis following influenza infection (IAPA) in immunocompetent patients (26). The increased risk of developing aspergillosis in patients with preceding influenza can be partially attributed to viral-induced epithelial disruption, the first line of host defense against fungal infections. However, recent evidence suggests that the second (phagocytosis and the killing of *Aspergillus* conidia by phagocytes) and third lines (extracellular mechanisms, mediated by neutrophils, to kill the *Aspergillus*) of the antifungal host responses are also impaired in patients with IAPA (1, 27). IAPA has also been documented to provoke a severe inflammatory response, resulting in a cytokine storm within the lung tissue (28). IAPA has been described for decades and has been increasingly recognized since the 2009 H1N1 influenza pandemic. Notably, *Aspergillus* species are also implicated in causing super-infection during SARS-CoV-2 (COVID-19) infections (29, 30), underscoring the importance of studying viral–fungal super-infections. Additionally, studies suggest that the hyperinflammatory responses driven by systemic cytokines in COVID-19 patients also contribute to CAPA (31).

The Type 17 cytokine family is recognized for its pivotal role in fostering protective immunity against a spectrum of pathogens (32). Previous research has substantiated the involvement of IL-17 in the context of viral–bacterial super-infections (15). The IL-17 pathway has also promoted *Aspergillus* clearance within pulmonary tissues (10–12). Mechanistically, the protective role for IL-17 is mediated by the recruitment of neutrophils through chemokine signaling and the production of antimicrobial peptide (AMP) production (33). Our current study aims to delineate the specific roles of IL-17 and Type 17 immune-associated cytokines and antimicrobial peptides during IAPA.

Our results demonstrate that a preceding influenza infection impairs Type 17 immunity during IAPA. This confirms and expands upon findings recently published by Lee et al. and Seldeslachts et al. (34, 35). We demonstrate that both the gene expression and the protein quantification of key Type 17 immune cytokines, including IL-17, IL-22, and IL-23, are decreased during IAPA compared to singular *Aspergillus* infection. IL-17, a pro-inflammatory cytokine, plays an essential role in fungal infections by recruiting neutrophils and other immune cells to the site of infection and by inducing the production of antimicrobial peptides (36). Additionally, IL-17 synergistically collaborates with IL-22 for a robust immune response against fungal infection (37), and their inhibition can lead to a compromised ability to control fungal infections (10). Decreased IL-17 production was also observed by Lee et al. (34), using a similar murine model of IAPA; however, we also showed reduction in other Type 17-immune-associated cytokines and antimicrobial peptides. IL-1β, a pro-inflammatory cytokine critical to Type 17 immunity, trends toward reduction in IAPA compared to singular *A. fumigatus* challenge. IL-1β has been reported to induce neutrophil and macrophage recruitment to the lungs during microbial invasion (38) and stimulates endothelial adhesion molecules, different cytokines and chemokines, and the Th17 adaptive immune response (39). Our results align with the previous findings of downregulation of *IL1B* that was observed in humans during IAPA (27). It is also important to highlight that during IAPA, the functional impairment of neutrophils and macrophages is a critical factor in the progression of the disease along with the leukocyte numbers (27, 40). Defective immune responses can allow the fungus to thrive even when leukocyte numbers are restored. Furthermore, in our murine model of IAPA, the impaired production of IL-17 and IL-22 is also associated with reduced levels of antimicrobial peptides. Antimicrobial peptides induced by IL-17 and IL-22 cytokines play an essential role in limiting fungal invasion. The role of IL-17-induced antimicrobial peptides in fungal infections is well documented, as IL-17RA^−/−^ mice exhibit heightened susceptibility to fungal pathogens, which is associated with impaired neutrophil recruitment and decreased AMP production (41). Besides direct pathogen killing, these peptides enhance the epithelial barrier function and promote neutrophil recruitment to the infection sites, providing a crucial first-line defense against *Aspergillus*.

CD4^+^ T cells and γδ T cells are recognized as the primary sources of IL-17 during fungal infections. Given the sharp decline in IL-17 levels observed in co-infected mice, it was crucial to assess how co-infection affects these parent cell populations. Our results demonstrated a selective reduction in IL-17-producing γδ T cells in super-infected mice compared to those infected only with *A. fumigatus*. Given the well-established role of γδ T cells as early responders to fungal infections, this decrease suggests that super-infection may impair the early innate immune response, potentially compromising fungal clearance at the initial stages (42). γδ T cells are known to produce IL-17 rapidly in response to pathogens, which is critical for neutrophil recruitment and antifungal defense (43). Interestingly, the abundance of IL-17^+^ CD4^+^ T cells remained unchanged between groups, suggesting that the adaptive immune response, in terms of IL-17 production, may not be significantly altered by super-infection at this early time point. When interpreting these results, it is important to consider the timing of mouse harvesting post-*A. fumigatus* infection. Specifically, the analysis was conducted at a 48-h time point post-infection. At this early stage, the immune response is still evolving, with distinct subsets of immune cells playing varying roles. In particular, it is noted that there were more innate γδ T cells present at this time point, with T cells potentially infiltrating at the later stages of infection. Previous studies have shown that CD4^+^ T cells play a more prominent role in the later stages of fungal infections, with IL-17 production peaking after the initial fungal clearance (43). These findings suggest that γδ T cells play a more critical role in early IL-17-mediated defense during super-infection, while the role of CD4^+^ T cells may become more evident in the later stages of infection. Notably, Seldeslachts et al. observed different results in their IAPA model with decreased production of IL-17 by both γδ and CD4^+^ T cells; however, this may be model-dependent with infectious challenges and harvest time point differences between the two models (35). Overall, there is likely inhibition of IL-17 production by both cell types, with early inhibition of γδ T cells and later inhibition of CD4^+^ T cells.

Despite the observed reduction in Type 17 immunity during IAPA, restoration of the components of the IL-17 signaling pathway, both upstream and downstream of IL-17, did not lead to improved fungal clearance. Irrespective of using both recombinant IL-17 protein and an adenoviral vector expressing IL-17, neither approach successfully reduced the fungal burden in IAPA. Although adenovirus expressing IL-17 provides prolonged IL-17 expression, it still did not achieve the anticipated enhancement of fungal clearance. Similarly, recombinant IL-1β and IL-23, upstream regulators of Type 17 immunity, increased the expression of IL-17, but a reduction in fungal burden was not observed. Additionally, the exogenous replacement of IL-17 did not enhance fungal clearance in singular *Aspergillus* infection in our studies. The role of Type 17 cytokines in modulating host immune responses to *A. fumigatus*, particularly through the induction of IL-17A, has been a focus of previously published studies. *In vitro* experiments have revealed that recombinant IL-23 has the capacity to induce both IL-17A and IL-22 production in murine lung cells and that the inhibition or deletion of IL-17A or IL-22 results in higher *Aspergillus* burden (10–12). Although the loss of IL-17A and/or IL-22 can result in higher *Aspergillus* burden, the addition of exogenous cytokine may not directly decrease the fungal burden. While Type 17 cytokines are recognized as critical mediators of antifungal immunity, particularly in neutrophil recruitment and epithelial barrier integrity, upregulation of Type 17 immunity alone does not enhance fungal resolution in our murine IAPA model. Our findings demonstrate that although Type 17 immunity may play a role during IAPA, restoration of this singular pathway does not restore fungal clearance, highlighting that other mechanisms may play a more pivotal role in IAPA pathogenesis.

Finally, the IL-17/IL-22-associated antimicrobial peptides, Reg3β and Reg3γ, are downregulated during IAPA compared to *Aspergillus* infection alone; however, the exogenous administration of these proteins did not alter the fungal burden in our model. While our findings suggest a potential role for an impaired Type 17 response in IAPA, direct evidence establishing this as the primary defect is lacking. This suggests that additional factors beyond IL-17 signaling are essential for effective immune responses against *A. fumigatus* in the context of viral co-infection, indicating a more complex immunosuppressive environment during IAPA that cannot be fully rescued by IL-17 alone. Seldeslachts et al. recently showed that mice lacking IFN-γ are both protected from IAPA and have restored Type 17 immunity (35). However, they also observed other immune changes in their model that could be essential to fungal clearance, as we have demonstrated that IAPA cannot be fully rescued by IL-17 alone. The immune response during viral/fungal super-infection is complex, and there are likely multiple drivers to the severe clinical consequences seen during IAPA. Alternatively, there may be other possible mechanisms, such as influenza-mediated suppression of phagocyte antifungal defenses, which warrant further investigation.

In contrast to prior studies that showed that restoration of Type 17 immune pathway components rescued bacterial clearance during post-influenza bacterial super-infection (15, 16), the current study indicates that restoration of IL-17 signaling alone is not sufficient to reduce the fungal burden in a murine IAPA model. These findings suggest that IL-17 may play a protective role but not a restorative role during IAPA. Notably, as the immune response is dynamic during viral and fungal infections, augmentation of Type 17 signaling at other time points during IAPA may produce different results. Interestingly, the trend of downregulation of IL-1β in our mouse model is consistent with the findings in human patients with IAPA versus influenza alone (27). This consistency between our findings in mice and human patients strengthens the validity of our results and suggests that the observed decrease in cytokines is a robust effect that is relevant across species. However, a limitation of this study is the exclusive use of male mice, which may not fully capture potential sex-specific differences in immune responses and disease progression, and therefore further studies incorporating both male and female mice are warranted to address this gap. Importantly, a key conclusion from these studies is that immune regulation during post-influenza fungal super-infection and post-influenza bacterial super-infection may be similar but likely do not have the same mechanisms. It underscores the need for additional studies to better understand the immune mechanisms that increase susceptibility to fungal infection during influenza and how delineation of the specific cell types and immune pathways that are necessary for fungal host defense during viral infection may lead to future therapeutics.

## Data Availability

The original contributions presented in the study are included in the article/**Supplementary Material**. Further inquiries can be directed to the corresponding author.
