# Supplementary material for: Restoration of Type 17 immune signaling is not sufficient for protection during influenza-associated pulmonary aspergillosis
Source: Front Immunol. 2025 Jan 30;16:1529849. doi: 10.3389/fimmu.2025.1529849 (PMC11821594; doi:10.3389/fimmu.2025.1529849)
Supplement: Supplementary file 1 [file DataSheet1.docx]

Supplementary Material

**Figure S1**: Relative expression of IL-17 during IAPA compared to singular infection with aspergillus. Wild-type C57BL/6 mice were infected with influenza virus, followed by infection with *A. fumigatus* resting conidia six days later. IL-17 expression was assessed at 24, 48, 72, 96 and 120 hours post-fungal infection. Data represent the mean ± SEM of biological replicates. Statistical significance was determined using two-way ANOVA with Šídák's multiple comparisons test.

**Figure S2**: Bronchoalveolar lavage (BAL) cell counts (**A**) and BAL differentials (**B**) were measured in wild-type mice infected with influenza A/PR/8/34 H1N1 (Flu) and *A. fumigatus* ATCC42202 resting conidia (AF). The administration of exogenous IL-17 adenovirus (IL-17) did not significantly affect BAL cell counts or any of the differential cell populations tested. Statistical significance of each cell was assessed using an unpaired Student’s t-test (A) and two-way ANOVA with Šídák's multiple comparisons test (B). Each experiment was independently performed twice, and the data presented are combined from these experiments.

**Figure S3**: Measurement of Bronchoalveolar lavage (BAL) cell counts in absence (PBS control) and presence of (**A**) different cytokines (IL-1β and IL-23/IL-1β) and (**C**) antimicrobial peptides (Reg3β or Reg3γ) in wild-type mice infected with influenza A/PR/8/34 H1N1 and *A. fumigatus* ATCC42202 resting conidia. The administration of IL-1β and IL-23/IL-1β and antimicrobial peptides, resulted no significant change in BAL cell counts in the context of IAPA. In addition, the impact on differential cell populations was recorded in presence of (**B**) IL-1β and IL-23/IL-1β and (**D**) Reg3β or Reg3γ. None of the externally provided factors except IL-1β significantly affect the differential cell populations tested. This experiment was independently repeated twice, with data presented as means ± SEM. Statistical significance was determined using an ordinary one-way ANOVA or two-way ANOVA with Tukey’s multiple comparisons test, with ****p* < 0.0005.
